# Microbiological findings and antibiotic treatment in community-acquired pneumonia: a retrospective cohort study

**DOI:** 10.1186/s12879-025-12510-0

**Published:** 2026-01-29

**Authors:** Sabisan Shanmuganathan, Jette Brommann Kornum, Niels Henrik Bruun, Lone Hagens Mygind

**Affiliations:** 1 Department of Infectious Diseases, Aalborg University Hospital, Aalborg, 9000 Denmark; 2 Department of Clinical Microbiology, Aalborg University Hospital, Aalborg, 9000 Denmark; 3 Department of Research Data and Biostatistics, Aalborg University Hospital, Aalborg, 9000 Denmark

**Keywords:** Pneumonia, CAP, Guideline adherence, Microbiological aetiology, *Haemophilus influenzae*

## Abstract

**Background:**

National and regional guidelines are regularly updated to ensure the correct diagnosis and treatment of pneumonia while minimizing unnecessary antibiotic use. However, recent microbiological studies have raised concerns about the recommendations in Danish guidelines. This study aimed to describe the aetiology, empirical antibiotic treatment, and adherence to guidelines in the management of hospitalised patients with community-acquired pneumonia (CAP).

**Methods:**

This retrospective cohort study included all adults hospitalised with CAP at the Emergency Department of Aalborg University Hospital, Denmark, over a one-year period from November 2021 to October 2022. Hospital records were reviewed, and the microbiological data and the antibiotic therapy were analysed.

**Results:**

A total of 366 patients were included in the study. The most frequently identified pathogen was *Haemophilus influenzae* (25%), followed by influenza A (22%), *Staphylococcus aureus* (9%), respiratory syncytial virus (9%) and *Streptococcus pneumoniae (8%)*. *H. influenzae* was the dominant bacterial pathogen in both patients with COPD and without COPD. *S. aureus* was among the most commonly detected pathogens in patients with a CURB-65 score of 3–5 (22%). Regarding antibiotic treatment, 41% of the patients did not receive the recommended therapy. Non-adherence to the guidelines was primarily driven by the overuse of broad-spectrum antibiotics. At admission time, the most commonly prescribed empirical antibiotics were amoxicillin/clavulanic acid (33%), piperacillin/tazobactam monotherapy (23%) and penicillin monotherapy (21%), respectively.

**Conclusion:**

Our study found that *H. influenzae* was the most frequently detected bacterial pathogen identified in both patients with and without COPD hospitalised with CAP. These findings highlight the need to reconsider the empirical treatment recommendations in the Danish guidelines. Amoxicillin/clavulanic acid was the most commonly prescribed empirical antibiotic. However, a substantial proportion of patients did not receive guideline-adherent treatment. Broad-spectrum antibiotic overuse was the main issue.

**Supplementary Information:**

The online version contains supplementary material available at 10.1186/s12879-025-12510-0.

## Background

Pneumonia is one of the leading causes of hospitalisation. In Denmark, approximately 29,000 people are hospitalised with pneumonia each year [1]. The disease is associated with increased morbidity and mortality, and community-acquired pneumonia (CAP) is the most common infectious cause of death in Europe [2, 3].

Lower respiratory tract infections, including pneumonia are among the primary reasons for antibiotic prescriptions in Europe [3]. However, antibiotic treatment is not without risks due to the adverse effects and antibiotic resistance which pose a growing global health threat. Broad-spectrum antibiotics, in particular, are key drivers of resistance [4, 5]. According to the World Health Organization (WHO), antibiotic resistance is responsible for an estimated 33,000 deaths annually in Europe [6].

To address this issue, several countries have implemented guidelines for antibiotic use. Adhering to these guidelines not only reduces broad-spectrum antibiotic consumption but also shortens hospital stays, lowers in-hospital mortality, and improves cost-effectiveness [7, 8]. Despite these benefits, studies indicate that guideline adherence in hospitals remains a challenge [9, 10].

In Denmark and other Scandinavian countries, the empirical treatment for CAP depends on the pneumonia severity which should be assessed using the CURB-65 scoring system (confusion, urea, respiratory rate, blood pressure, age $\:\ge\:$65 years). Mild CAP is empirically treated with penicillin primarily to target *Streptococcus pneumoniae*, as it is considered the most frequent bacterial cause of CAP [3, 11, 12]. However, other countries have different recommendations for the antibiotic treatment of CAP. In the American, British, and German guidelines, a broader-spectrum antibiotic, amoxicillin, is recommended as the empirical treatment for mild CAP [13–15]. In Spain, monotherapy with quinolone is the recommended empirical antibiotic for hospitalised patients with CAP [16]. One reason for this difference in recommendations between the Scandinavian countries and others may be variations in the bacterial aetiology and antibiotic resistance. The penicillin resistance of *S. pneumoniae* is 22.3% in Spain, whereas, it is only 0.6% in Denmark [3, 17].

Although, the empirical treatment of mild CAP in Denmark is penicillin to ensure initial coverage of *S. pneumoniae*, a recent study by Fally et al. (2021) reports *Haemophilus influenzae* as the most frequent bacterial aetiology of CAP in Denmark [18]. However, a Danish study by Thønnings et al. shows that treatment of *H. influenzae* bacteremia with benzylpenicillin is associated with increased 30-day mortality compared to treatment with aminopenicillins or cefuroxime [19]. These findings raise concerns about the recommendations in the Danish guidelines for CAP management due to potential penicillin resistance in *H. influenza*e.

Hence, this study aimed to evaluate empirical antibiotic treatment and guideline adherence in hospitalised patients with community-acquired pneumonia (CAP), including associated microbiological and clinical outcomes, with subgroup analyses by chronic obstructive pulmonary disease (COPD) status and CURB-65 severity.”

## Methods

### Study design and participants

This single-centre cohort study included all patients $\:\ge\:$18 years admitted with CAP to the Emergency Department (ED) of Aalborg University Hospital, Denmark, in a 12-month period between November 1, 2021, and October 31, 2022. A 12-month period was chosen to account for potential seasonal variations. CAP was defined by the presence of a new infiltrate on chest radiograph accompanied by at least one of the following symptoms or clinical findings: body temperature $\:>$38.0 °C, cough, exacerbated expectoration, dyspnoea, auscultatory rales or percussive dullness [11, 20].

Patients were also included if they were admitted for other conditions, but simultaneously met the criteria for pneumonia. If a patient was hospitalised with pneumonia more than once during the 12-month period, only the first admission was included to maintain the independence of the data.

Exclusion criteria included pneumonia that developed $\:\ge\:$48 hours after hospital admission or intubation, hospitalisation within the past 28 days, COVID-19, active tuberculosis, immunocompromised status or if aspiration pneumonia was suspected by the attending physician. Patients were classified as immunocompromised if they were HIV-positive, had received chemotherapy or immunomodulating drugs within the past 28 days, had received corticosteroids ($\:\ge\:$20 mg prednisolone-equivalent per day for more than 14 days), had neutrophil granulocytes counts $\:<$1.0 × 10^9^/L, were immunocompromised after an organ transplantation or were splenectomised.

Moreover, patients who were directly transferred to intensive care unit (ICU), other wards or other hospitals were not included. 

### Data collection

A physician involved in the current study identified all patients who had a chest radiograph performed in the ED on a daily basis. This was done by reviewing the records of all hospitalised ED patients in the electronic health record system. The radiological interpretation of the chest x-ray determined the presence of a newly developed infiltrate. Once patients with a new infiltrate were identified, their hospital records were reviewed to exclude those who did not meet the criteria for CAP.

Data were collected directly from the hospital records by a physician involved in the study. The data were managed in a database in the Research Electronic Data Capture (REDCap) software platform hosted in the North Denmark Region [21, 22]. Microbiological findings were reported from the following methods: blood cultures; sputum cultures; tracheal aspirate cultures; bronchoalveolar lavage (BAL) fluid cultures; PCR detection of atypical pathogens (*Chlamydia pneumoniae*, *Chlamydia psittaci*, *Legionella pneumophila* and *Mycoplasma pneumoniae*) from sputum, tracheal aspirate and BAL samples; urinary antigen tests for *S. pneumoniae* (PUAT) and *L. pneumophila* (LPUAT); and PCR detection of respiratory viruses (influenza A and B and respiratory syncytial virus (RSV)) from oropharyngeal swab samples. The samples were analysed by the local Department of Clinical Microbiology. If the same microbiological pathogen was identified across multiple tests, only one result was recorded.

The adherence of empirical antibiotic therapy to the antibiotic guidelines was evaluated based on the regional guidelines of the North Denmark Region for CAP. According to these guidelines, the choice of empirical antibiotic treatment was determined by the severity of pneumonia. Mild pneumonia was defined as a CURB-65 score of 0–2, moderate to severe pneumonia as a CURB-65 score of 3–5, and severe pneumonia as a CURB-65 score of 3–5 with at least one of the following: involvement of more than one pulmonary lobe, severe hypoxia with an oxygen saturation below 92% or sepsis [11, 20]. If the CURB-65 score was not documented by the attending physician, it was calculated from the information available in the hospital records. In cases where the distribution of infiltrates on the chest radiograph was not reported, a single infiltrate was assumed. The presence of sepsis was assessed using the quick sequential organ failure assessment (qSOFA) score (respiratory rate, blood pressure, confusion; sepsis if $\:\ge\:$2) [23]. Additionally, when evaluating guideline adherence, the attending physician’s tentative diagnosis was also taken into account. If the treating physician suspected another concurrent infection in addition to CAP, the treatment was considered adherent to the guidelines, even if antibiotics other than those recommended in the pneumonia guidelines were initiated. However, if no antibiotics were administered despite meeting the pneumonia criteria, the case was considered non-adherent to the guidelines.

Guideline adherence was assessed according to the North Denmark Region guideline for the treatment of CAP [20]. In the North Denmark Region, the recommended empirical treatment of community-acquired pneumonia (CAP) varies according to disease severity and the presence of chronic obstructive pulmonary disease (COPD). For patients with mild CAP, penicillin 1 MIU is advised, while moderate CAP is treated with penicillin 2 MIU in combination with clarithromycin. Severe CAP requires broader coverage with piperacillin/tazobactam together with clarithromycin. In patients with COPD, amoxicillin/clavulanic acid is recommended instead of penicillin in mild cases, and in moderate cases it is combined with clarithromycin. For severe CAP in COPD patients, piperacillin/tazobactam plus clarithromycin remains the recommended regimen (see Supplementary Table S1).

### Outcome

The primary outcome of the study was the empirical antibiotic treatment of patients with CAP at admission time. In our study, empirical therapy at admission was defined as the first antimicrobial treatment initiated in the emergency department. Secondary outcomes were the microbiological aetiology, and whether the patients hospitalised with CAP received antibiotic treatment in adherence to the regional guidelines. When analysing the guideline adherence, length of stay, 30-day all-cause rehospitalisation, and 30-day all-cause mortality were compared between the guideline-adherent population and the non-adherent population.

A subgroup analysis was performed comparing the microbiological aetiology of CAP in patients with and without COPD, and in patients with a CURB-65 score of 0–2 versus 3–5, as the regional guidelines of the North Denmark Region distinguish between these populations in their recommendations for empirical treatment based on differences in prevalent pathogens and resistance patterns.

### Statistical methods

All statistical analyses were performed in Stata Statistical Software: Release 17. College Station, TX: StataCorp LLC. Results were reported as counts (%) if categorical data and as medians with interquartile ranges (IQR) if continuous data. The chi-square test was used for categorical data. There was no comparison of continuous data. The relationship between guideline adherence and length of stay, 30-day all-cause rehospitalisation, and 30-day all-cause mortality, respectively, was evaluated using Poisson regression with robust variance estimation, and relative risks (RR) with corresponding 95% confidence intervals (95% CI) were reported. A P-value < 0.05 was considered statistically significant.

### Ethics

The study was performed in accordance with the Helsinki declaration. According to the Danish Act on scientific ethical treatment of health science research projects, register based studies do not require ethics committee approval or informed consent [24]. The processing of personal data was approved by the Region of Northern Denmark with the ID-number K2022-046 and listed in the internal record cf. art 30 of The EU General Data Protection Regulation.

## Results

### Patient characteristics

During the 12-month study period, 366 patients with CAP were included. Patients with chronic obstructive pulmonary disease (COPD) comprised 43% of the study population. The majority of patients had a CURB-65 score $\:\le\:$2 (75%) (Table 1). However, the CURB-65 score was documented in only 12% of the hospital records. For the overall study population, the median length of stay was 4 days (IQR 3–7), and the 30-day all-cause mortality was 15%. The 30-day all-cause rehospitalisation was 18%, with 38% of these patients meeting the criteria for a new pneumonia episode.

**Table 1:** Patient characteristics

Characteristic	Mild CAP	Moderate CAP	Severe CAP	Total
	***N*** **= 274**	*N* = 24	*N* = 68	*N* = 366
Age, median (IQR)	76 (69–82)	82 (72–92)	83 (77–88)	78 (70–84)
Gender, female	134 (49%)	10 (41%)	34 (50%)	178 (49%)
Nursing home resident	39 (14%)	7 (29%)	30 (44%)	76 (21%)
Co-morbidities				
COPD^1^	119 (43%)	9 (38%)	29 (43%)	157 (43%)
Asthma	15 (5%)	0 (0%)	2 (3%)	17 (5%)
Other chronic respiratory diseases^2^	21 (8%)	0 (0%)	3 (4%)	24 (7%)
Chronic kidney disease	26 (9%)	1 (4%)	16 (24%)	43 (12%)
Chronic heart disease	23 (8%)	0 (0%)	10 (15%)	33 (9%)
Chronic liver disease	8 (3%)	0 (0%)	1 (1%)	9 (2%)
Neoplastic disease	21 (8%)	3 (13%)	10 (15%)	34 (9%)
Diabetes mellitus	57 (21%)	3 (13%)	17 (25%	77 (21%)
Smoking status				
Never smoked	60 (22%)	5 (21%)	12 (18%)	76 (21%)
Currently smoking	63 (23%)	6 (25%)	11 (16%)	80 (22%)
Ex-smoker	118 (43%)	8 (33%)	20 (29%)	146 (40%)
Not reported	33 (12%)	6 (25%)	25 (37%)	64 (17%)
Alcohol status				
None excessive alcohol consumption	203 (74%)	15 (63%)	28 (41%)	246 (67%)
Excessive alcohol consumption^3^	21 (8%)	0 (0%)	3 (4%)	24 (7%)
Former excessive alcohol consumption	12 (4%)	1 (4%)	7 (10%)	20 (5%)
Not reported	38 (14%)	8 (33%)	30 (44%)	76 (21%)
Chest radiograph				
Single infiltrate	148 (54%)	16 (67%)	17 (25%)	181 (49%)
Infiltrates in > 1 pulmonary lobe	98 (36%)	0 (0%)	46 (68%)	144 (39%)
Distribution of infiltrates not reported	28 (10%)	8 (33%)	5 (7%)	41 (11%)

Data are n (%), unless otherwise indicated.

1) Chronic obstructive pulmonary disease

2) Interstitial lung disease, pulmonary cancer, lobectomy, asbestosis, bronchiectasis, emphysema, chronic bronchitis, sleep apnoea

3) Excessive alcohol consumption: defined as more than 10 standard drinks per week

### Microbiological findings

**Fig. 1:**
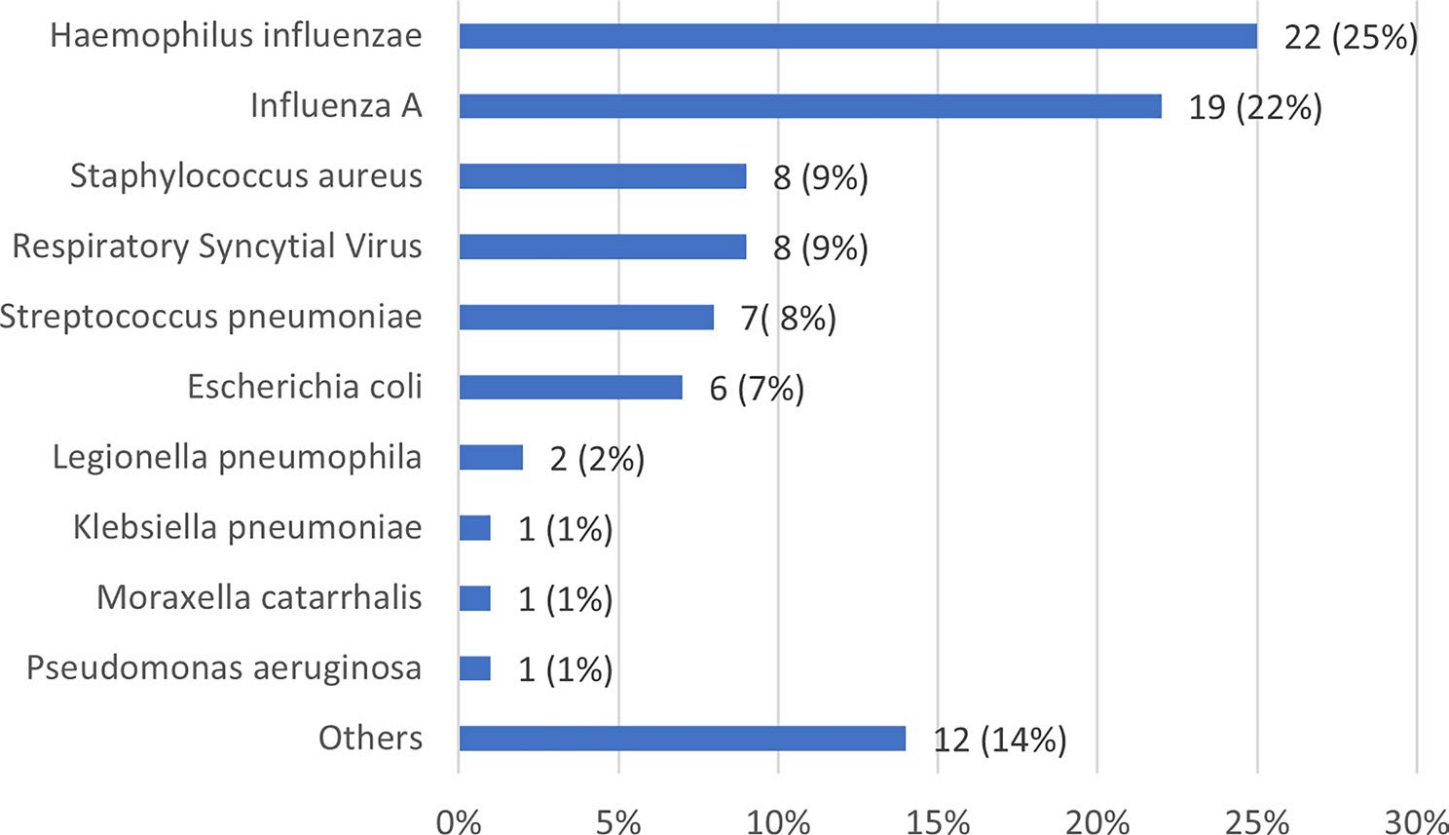
Distribution of microbiological findings. Data are n (%). Others: Enterobacteriaceae species (*n* = 5), yeast species (*n* = 2), aerobic gram-negative bacilli (*n* = 2), *Enterococcus faecalis* (*n* = 1), *Streptococcus mitis* (*n* = 1), *Abiotrophia defectiva* (*n* = 1)

Almost all patients underwent at least one microbiological test (98%). PCR testing for respiratory viruses (91%) and blood cultures (87%) were performed in most of the patients. However, sputum cultures, tracheal aspirate cultures and BAL cultures were only performed in 36% of the patients, while PCR testing for atypical pathogens was only conducted in 13% (see Supplementary Table S2). Of those with at least one microbiological test, 22% had positive findings. The distribution details for the microbiological findings are shown in Fig. 1. The most common pathogen identified was *H. influenzae*, found in 25% of all patients with positive findings and among 6% of total population N. The second most frequently detected pathogen was Influenza A ( *n* = 22% an *N* = 2%), with all cases of influenza A occurring between March and May 2022. Other pathogens included RSV (*n* = 9%, *N* = 2%), *Staphylococcus aureus* (*n* = 9%, *N* = 2%) and *Streptococcus pneumoniae* (*n* = 8%, *N* = 2%). In total, four patients (*n* = 5%, *N* = 1%) had a mixed bacterial-viral infection. Regarding *Escherichia coli*, all six cases (100%) were detected in blood cultures, with only one case found in sputum cultures.

### Microbiological findings and subgroups

*H. influenzae* was the most common bacterial pathogen identified in both patients with COPD (32%) and patients without COPD (24%). *S. pneumoniae* was detected in 13% of patients with COPD and 6% of patients without COPD.

In terms of CURB-65 score, *H. influenzae* (29%) was the most frequently identified pathogen among patients with a score of 0–2. In contrast, *S. aureus* (22%) was identified as frequently as *H. influenzae* (22%) among patients with higher severity scores (CURB-65 score of 3–5) (Table 2).

**Table 2:** Microbiological findings and subgroups positives tests (The proportion among tests n (%)/ and the proportion among the cohort N(%))

Subgroups	*n* (%) / *N* (%)
**Patients with COPD**	157 (43%)
Positive test	31 (20%)
* H. influenzae*	10 (32%) / (6%)
Influenza A	6 (19%) / (4%)
* S. pneumoniae*	4 (13%) / (1%)
**Patients without COPD**	209 (57%)
Positive test	49 (23%)
Influenza A	13 (27%) / (6%)
* H. influenzae*	12 (24%) / (6%)
Respiratory syncytial virus	7 (14%) / (3%)
* S. aureus*	5 (10%) / (2%)
* E. coli*	4 (8%) / (2%)
**CURB-65 score**	
0–2	274 (75%)
Positive test	62 (23%)
* H. influenzae*	18 (29%) / (7%)
Influenza A	17 (27%) / (6%)
Respiratory syncytial virus	7 (11%) / (3%)
* S. pneumoniae*	5 (8%) / (2%)
* E. coli*	4 (6%) / (1%)
* S. aureus*	4 (6%) / (1%)
3–5	92 (25%)
Positive test	18 (20%)
* H. influenzae*	4 (22%) / (4%)
* S. aureus*	4 (22%) / (4%)

Only findings 4 are reported

### Antibiotic treatment

A total of 94% of patients received antibiotics upon admission. The most commonly prescribed empirical antibiotic was amoxicillin/clavulanic acid (33%) followed by piperacillin/tazobactam monotherapy (23%) and penicillin monotherapy (21%) (Table 3). In total, 78 patients (21%) received treatment for a concomitant condition at presentation.

**Table 3:** Antibiotic treatment

Outcome	Study population *N* = 344 (94%)
**Most prescribed antibiotics at admission time**	
Amoxicillin/clavulanic acid	115 (33%)
Piperacillin/tazobactam monotherapy	78 (23%)
Penicillin monotherapy	71 (21%)
Ampicillin/gentamicin	25 (7%)
Cefuroxime monotherapy or combination therapy	17 (5%)
Penicillin and clarithromycin	13 (4%)
Piperacillin/tazobactam and clarithromycin	9 (3%)
Clarithromycin monotherapy	5 (1%)
Others^1^	11 (3%)

Data are n (%), unless otherwise indicated

1) Clarithromycin monotherapy, ciprofloxacin, roxithromycin, moxifloxacin, or meropenem

### Adherence to guidelines

Overall, 59% of patients received the correct empirical treatment in accordance with the regional guidelines. Patients with COPD and those with a CURB-65 score of 0–2 were more likely to receive the correct empirical antibiotic treatment compared to patients without COPD (RR 1.45, 95% CI 1.22–1.71) and those with a CURB-65 score of 3–5 (RR 1.69, 95% CI 1.30–2.22), respectively.

The main reason for non-adherence to the guidelines was the prescription of broad-spectrum antibiotics for cases of mild or moderate pneumonia (Table 4). No statistically significant differences were found in length of stay (RR 1.03, 95% CI 0.80–1.33), 30-day all-cause rehospitalisation (RR 1.39, 95% CI 0.84–2.28), and 30-day all-cause mortality (RR 1.02, 95% CI 0.51–2.03) between guideline-adherent and guideline-non-adherent patients (*P* > 0.05).

**Table 4:** Reasons for non-adherence to guideline

Reason	*n* (%)
Overtreatment with broad-spectrum antibiotics	59 (40%)
Undertreatment with narrow-spectrum antibiotics	25 (17%)
No antibiotics prescribed at admission time	22 (15%)
No clarithromycin added	17 (12%)
Wrong antibiotic class	10 (7%)
COPD treatment prescribed despite patient without COPD	8 (5%)
Non-COPD treatment prescribed despite patient with COPD	6 (4%)

## Discussion

### Key findings

Our study aimed to report the microbiological aetiology and antibiotic treatment of patients hospitalised with pneumonia. The findings revealed that *H. influenzae* was the most common bacterial pathogen identified in both patients with COPD and without COPD. The most commonly prescribed empirical antibiotic was amoxicillin/clavulanic acid, followed by piperacillin/tazobactam and penicillin. In total, 41% of the study population did not receive correct antibiotic treatment in accordance with regional guidelines at the time of admission. The primary reason for non-adherence to these guidelines was overtreatment with broad-spectrum antibiotics.

### Comparison with the literature

#### Microbiological findings

Consistent with our study, a Danish cohort study comprising 2,264 patients with CAP published in 2021 by Fally et al. also identified *H. influenzae* as the most frequent pathogen. In our study, 25% of microbiological findings were *H. influenzae*, compared to 24% in Fally et al.’s study [18]. Furthermore, a study by Gadsby et al. of 326 patients with CAP from the UK and using comprehensive molecular testing, also found *H. influenzae* as the dominant pathogen, but at a higher frequency of 40%. Gadsby et al. identified rhinovirus as the most common viral pathogen, which was not tested for in our study. Both Fally et al. and Gadsby et al. reported influenza A in 9% and 5% of the cases, respectively, while our study found it in 22%. This notable difference could be attributed to seasonal variations or differences in study timeframes.

Another difference was the detection of *S. pneumonia.* Fally et al. and Gadsby et al. reported a higher proportion of *S. pneumoniae* (20% and 36%, respectively) compared to our study, where it comprised only 8% [18, 25]. This difference may be explained by the widespread pneumococcal vaccination in Denmark and the implementation to the Danish childhood vaccination programme. Since 2017, approximately 80% of the citizens above 65 years have received the pneumococcal polysaccharide vaccine (PPSV23) with adherence increasing drastically during the COVID-19 pandemic [26]. This could explain the difference as Fally et al. and Gadsby et al. reported data from before the COVID-19 pandemic.

Nevertheless, several previously published studies from Western countries, including Scandinavian countries, reported *S. pneumoniae* as the dominant bacterial pathogen of CAP [3, 27–35]. Although, the studies were published recently, many of them reported data from before 2018. Since our data was collected some years later, a shift in the microbiological findings could have occurred meanwhile. Furthermore, our data was collected during an ongoing COVID-19 pandemic which could have an influence on the bacterial aetiology of CAP. Besides, two of the studies investigated the aetiology of CAP over time and reported an increasing prevalence of *H. influenzae* contemporary with a decreasing prevalence of *S. pneumoniae* [36, 37]. Taking this course into account with our findings, it could indicate that *H. influenzae* will have a bigger impact in the near future, if not already.

Surprisingly, *S. aureus* was the second-most commonly identified bacterium in our study, a finding that contrasts with the results of Fally et al. and Gadsby et al., who both identified *S. pneumoniae* as the second-most common bacterium. This might be explained by the higher frequency of influenza A in our study, as secondary bacterial pneumonia caused by *S. aureus* is often linked to influenza A infection, particularly in nasal carriers of *S. aureus* [38].

However, influenza A was solely detected during a two-month period in the spring in our study. This was consistent with a study from Norway, where the viral findings varied considerably during the year with a peak during winter and spring [32].

We also found a relatively high proportion of *E. coli* in blood samples. It is important to consider whether *E. coli* detected in blood was the actual aetiology of the pneumonia or if it was found due to a co-infection, for instance a urinary tract infection. This ambiguity also applies to other bacterial pathogens, such as certain Enterobacteriaceae species.

### Microbiological findings and subgroups

As in patients with COPD, *H. influenzae* was also the most frequently identified pathogen amongst patients without COPD. This finding is in conflict with the Danish guidelines, which recommend penicillin for mild or moderate CAP in patients without COPD. A Norwegian study by Waagsbø et al. found *S. pneumoniae* as the dominant pathogen in patients without COPD [39]. A possible explanation for this difference is that some patients in our study may have undiagnosed COPD, given that 62% of the study population were current or former smokers, but only 43% had a formal COPD diagnosis.

Regarding the CURB-65 score, *S. aureus* was frequently identified in patients with a score of 3–5, which aligns with previous studies that show CAP caused by *S. aureus* is linked to greater illness severity and higher mortality rates [40, 41]. 

### Antibiotic treatment

Amoxicillin/clavulanic acid was the most prescribed empirical antibiotic, administered to 33% of patients in our study. This is consistent with the results of a multicentre study across 10 European Union countries by Blasi et al., which also reported amoxicillin/clavulanic acid as the most commonly used antibiotic [42].

Interestingly, only 25% of patients in our study received penicillin, despite it being the recommended empirical antibiotic for mild and moderate CAP in the Danish guidelines. In contrast, a Danish study by Egelund et al. found that 45% of patients received penicillin monotherapy. This difference may be explained by the higher proportion of patients with COPD in our study (43%) compared to Egelund et al.´s cohort (19%) [3]. Since amoxicillin/clavulanic acid is the recommended empirical treatment for mild or moderate CAP in patiens with COPD, this could explain the more frequent use of this antibiotic in our study. In another Danish study by Lorentzen et al., who had a similar proportion of patients with COPD and a comparable distribution of CURB-65 score, piperacillin/tazobactam was the most commonly prescribed antibiotic, which the authors attributed to diagnostic uncertainty. This reasoning may also explain the frequent use of piperacillin/tazobactam in our study [35].

### Adherence to guidelines

In total, 59% of patients in our study received empirical antibiotic treatment in accordance with regional guidelines, which is consistent with previously published studies (53–65%) [7, 43–48]. Some studies, however, report better outcomes, such as shorter length of stay, lower mortality, and reduced readmission rates, for patients receiving guideline-adherent therapy. For instance, McCabe et al. found that patients receiving guideline-concordant antibiotics had a reduced length of stay and in-hospital mortality [7], and Høgli et al. reported a decreased risk of 30-day readmission in patients receiving empirical penicillin G/V monotherapy [30]. This highlights the need to improve adherence to guidelines at our university hospital. Fally et al. demonstrated that tailored interventions could improve adherence, increasing guideline-concordant treatment from 59% to 74% [44].

### Strengths and limitations

Our study had both strengths and limitations. One major strength was the methodology used to include patients. Unlike Egelund et al., who identified pneumonia cases using ICD-10 codes, we required radiographic confirmation of infiltrates. This ensured inclusion of patients hospitalised primarily for co-infections or other comorbidities who also met the criteria for pneumonia, as well as cases that might otherwise have been overlooked due to more conspicuous comorbidities. This approach reduced the risk of misclassifying patients without true pneumonia and thereby strengthened the validity of our cohort [3].

A strength of the study is also its representative nature. Aalborg University hospital serves both urban and rural areas in the North Denmark region, providing highly specialised and basic medical care. Moreover, the hospital records were reviewed by only one person, which minimized variability due to differences in how physicians document patient information. The retrospective design allowed us to collect data independently of the study aims, reducing the risk of information bias.

However, a limitation of our study was the retrospective design itself, which depended on the completeness and accuracy of hospital records. For instance, the CURB-65 score was documented in only 12% of records, requiring us to calculate the score based on available data. This could introduce discrepancies between our classification of pneumonia severity and the physician’s initial assessment. Moreover, we only reported comorbidities documented in the hospital records, hence some comorbidities could be missing. In addition, microbiological testing was determined by the attending physicians as part of routine hospital practice, so not all pathogens associated with pneumonia may have been tested for, such as rhinovirus, which has been reported as a frequent pathogen in other studies [28, 32, 36]. Only 36% of patients underwent sputum, tracheal aspirate, or BAL cultures, which limits the ability to fully characterize the cohort’s pathogenic profile. Thus, the difference in microbiological findings compared to other studies could also be due to the differences in testing practices.

Another limitation was the small sample size compared to other studies, which may contribute to variability in the findings [18, 27, 28, 36]. Moreover, as this was a single-center study, the findings may have limited generalizability and could be affected by local practices and patient selection. Furthermore, patients who were directly transferred to ICU, other hospitals or other wards were not included, potentially leading to selection bias, as these patients may have been more severely ill. Finally, we did not account for antibiotics administered prior to hospital admission, which could influence the microbiological findings.

## Conclusion

In conclusion, our study found *H. influenzae* to be the dominant finding in patients with CAP, both with and without COPD. Amoxicillin/clavulanic acid was the most prescribed antibiotic drug. A total of 41% of patients did not receive empirical antibiotics in line with regional guidelines. The primary reason for non-adherence was overtreatment with broad-spectrum antibiotics. These findings, together with recent studies also identifying *H. influenzae* as the most common microbiological cause of CAP, raise the question of whether the empirical treatment recommendations in the Danish guidelines should be reconsidered to optimize patient outcomes and reduce the growing threat of antibiotic resistance. 

## Supplementary Information

Below is the link to the electronic supplementary material.

Supplementary Material 1

## Data Availability

The datasets generated and analysed during the current study are not publicly available due to Danish laws on personal data but can be available from the corresponding author on reasonable request.

## Abbreviations

BAL: Bronchoalveolar lavage fluid
CAP: Community-acquired pneumonia
COPD: Chronic obstructive pulmonary disease
ED: Emergency Department
ICU: Intensive care unit
IQR: Interquartile range
LPUAT: Urinary antigen tests for Legionella pneumophila
PUAT: Urinary antigen tests for Streptococcus pneumoniae
RR: Relative risk
RSV: Respiratory syncytial virus

## References

[1] Lungebetændelse. 2024. https://www.ssi.dk/sygdomme-beredskab-og-forskning/sygdomsleksikon/l/lungebetaendelse (accessed Dec, 2024).

[2] Welte T, Torres A, Nathwani D. Clinical and economic burden of community-acquired pneumonia among. Adults Europe. 2012;67:71–9. 10.1136/thx.2009.129502. 10.1136/thx.2009.129502 20729232

[3] Egelund GB, Jensen AV, Andersen SB, Petersen PT, Lindhardt BØ, von Plessen C et al. Penicillin treatment for patients with Community-Acquired pneumonia in denmark: a retrospective cohort study 2017;17:66. 10.1186/s12890-017-0404-8 10.1186/s12890-017-0404-8 PMC5397671 28427381

[4] Dellit TH, Owens RC, McGowan JE, Gerding DN, Weinstein RA, Burke JP, et al. Infectious diseases society of America and the society for healthcare epidemiology of America guidelines for developing an institutional program to enhance antimicrobial stewardship 2007;44:159–77. 10.1086/510393 10.1086/510393 17173212

[5] Tansarli GS, Mylonakis E. Systematic review and Meta-analysis of the efficacy of Short-Course antibiotic treatments for community-acquired pneumonia in adults 2018;62:e00635–18. 10.1128/AAC.00635-18 10.1128/AAC.00635-18 PMC6125522 29987137

[6] Antimicrobial resistance surveillance in. Europe 2022 – 2020 data. Copenhagen: WHO Regional Office for Europe: WHO Regional Office for Europe/European Centre for Disease Prevention and Control;; 2022.

[7] McCabe C, Kirchner C, Zhang H, Daley J, Fisman DN. Guideline-concordant therapy and reduced mortality and length of stay in adults with community-acquired pneumonia: playing by the rules 2009;169:1525–31. 10.1001/archinternmed.2009.259 10.1001/archinternmed.2009.259 19752411

[8] Egger ME, Myers JA, Arnold FW, Pass LA, Ramirez JA, Brock GN. Cost effectiveness of adherence to IDSA/ATS guidelines in elderly patients hospitalized for community. Aquired Pneumonia. 2016;16:34. 10.1186/s12911-016-0270-y. 10.1186/s12911-016-0270-y PMC4791973 26976388

[9] Tan YA, Scott IA. Guideline concordance in managing community-acquired pneumonia: room for improvement. 2022;14:79–88. 10.2147/CA.S377148

[10] Robinson HL, Robinson PC, Whitby M. Poor compliance with community-acquired pneumonia antibiotic guidelines in a large. Australian Private Hosp Emerg Department. 2014;20:561–7. 10.1089/mdr.2014.0064. 10.1089/mdr.2014.0064 24950058

[11] Retningslinjer for håndtering af voksne patienter. Indlagt med Pneumoni 1. udgave, 2021. Dansk Lungemedicinsk Selskab. Dansk selskab for infektionsmedicin; 2021.

[12] Lim W, van der Eerden MM, Laing R, Boersma W, Karalus N, Town G, et al. Defining community acquired pneumonia severity on presentation to hospital: an international derivation and validation study 2003;58:377–82. 10.1136/thorax.58.5.377 10.1136/thorax.58.5.377 PMC1746657 12728155

[13] Metlay JP, Waterer GW, Long AC, Anzueto A, Brozek J, Crothers K, et al. Diagnosis and treatment of adults with community-acquired pneumonia. An official clinical practice guideline of the American thoracic society and infectious diseases. Soc Am. 2019;200:e45–67. 10.1164/rccm.201908-1581ST. 10.1164/rccm.201908-1581ST PMC6812437 31573350

[14] Lim WS, Baudouin SV, George RC, Hill AT, Jamieson C, Le Jeune I, et al. BTS guidelines for the management of community acquired pneumonia in adults: update 2009 2009:iii1-55. 10.1136/thx.2009.121434 10.1136/thx.2009.121434 19783532

[15] Schmeck B, Steuder R, Pott H, et al. Update Ambulant Erworbene Pneumonie CME. 2022;19:8–11. 10.1007/s11298-022-2317-y.

[16] Menéndez R, Torres A, Aspa J, Capelastegui A, Prat C, Rodríguez de Castro F. Community-Acquired Pneumonia. New guidelines of the Spanish society of pulmonology and thoracic surgery. (SEPAR). 2010;46:543–58. 10.1016/S1579-2129(11)60008-6. 10.1016/j.arbres.2010.06.014 20832928

[17] Surveillance Atlas of Infectious Diseases 2021. 2021. https://atlas.ecdc.europa.eu/public/index.aspx?Dataset=27&HealthTopic=4 (accessed January 4, 2022).

[18] Fally M, Israelsen S, Anhøj J, Benfield T, Tarp B, Kolte L et al. The increasing importance of haemophilus influenzae in community-acquired pneumonia: results from a Danish cohort study 2021;53:122–30. 10.1080/23744235.2020.1846776 10.1080/23744235.2020.1846776 33231116

[19] Thønnings S, Østergaard C. Treatment of Haemophilus bacteremia with benzylpenicillin is associated with increased (30-day) mortality. 2012;12:153. 10.1186/1471-2334-12-153 10.1186/1471-2334-12-153 PMC3407763 22775981

[20] Mygind L, Kornum JB. Samfundserhvervet pneumoni hos voksne. 2022. https://pri.rn.dk/Sider/12931.aspx (accessed September 9, 2022).

[21] Harris PA, Taylor R, Thielke R, Payne J, Gonzalez N, Conde JG. Research electronic data capture (REDCap) a metadata-driven methodology and workflow process for providing translational research informatics support 2009;42:377–81. 10.1016/j.jbi.2008.08.010 10.1016/j.jbi.2008.08.010 PMC2700030 18929686

[22] Harris PA, Taylor R, Minor BL, Elliott V, Fernandez M, O’Neal L et al. The REDCap consortium: Building an international community of software platform partners 2019;95:103208. 10.1016/j.jbi.2019.103208 10.1016/j.jbi.2019.103208 PMC7254481 31078660

[23] Barbara P, Graziano C, Caputo W, Litvak I, Battinelli D, Hahn B. The quick sequential organ failure assessment (qSOFA) identifies septic patients in the out-of-hospital setting 2018;36:1022–6. 10.1016/j.ajem.2018.01.073 10.1016/j.ajem.2018.01.073 29426799

[24] Health TDmo. Act number 593 of 14/06/2011 - Act on scientific ethical treatment of health science research projects 2011. https://leap.unep.org/en/countries/dk/national-legislation/act-no-593-relative-ethical-medical-research

[25] Gadsby NJ, Russell CD, McHugh MP, Mark H, Conway Morris A, Laurenson IF, et al. Comprehensive molecular testing for respiratory pathogens in community. -Acquired Pneumonia. 2016;62:817–23. 10.1093/cid/civ1214. 10.1093/cid/civ1214 PMC4787606 26747825

[26] PPV-23 Vaccinationstilslutning. - Overvågning i tal, grafer og kort 2022. https://statistik.ssi.dk/sygdomsdata#!/?vaccination=24&sex=3&agegroup=4&landsdel=100&xaxis=Season&show=Graph&datatype=Vaccination (accessed December 31, 2022).

[27] Carugati M, Aliberti S, Reyes LF, Franco Sadud R, Irfan M, Prat C, et al. Microbiological testing of adults hospitalised with community-acquired pneumonia: an international study 2018;4:00096–2018. 10.1183/23120541.00096-2018 10.1183/23120541.00096-2018 PMC6174282 30474036

[28] Jain S, Self WH, Wunderink RG, Fakhran S, Balk R, Bramley AM, et al. Community-Acquired Pneumonia Requiring Hospitalization among U S Adults. 2015;373:415–27. 10.1056/NEJMoa1500245. 10.1056/NEJMoa1500245 PMC4728150 26172429

[29] Johansson N, Kalin M, Tiveljung-Lindell A, Giske CG, Hedlund J. Etiology of community-acquired pneumonia: increased Microbiological yield with new diagnostic methods 2010;50:202–9. 10.1086/648678 10.1086/648678 PMC7107844 20014950

[30] Høgli JU, Garcia BH, Svendsen K, Skogen V, Småbrekke L. Empirical prescribing of penicillin G/V reduces risk of readmission of hospitalized patients with community-acquired pneumonia in norway: a retrospective observational study 2020;20:169. 10.1186/s12890-020-01188-6 10.1186/s12890-020-01188-6 PMC7294665 32539706

[31] Røysted W, Simonsen Ø, Jenkins A, Sarjomaa M, Svendsen MV, Ragnhildstveit E, et al. Aetiology and risk factors of community-acquired pneumonia in hospitalized patients. Nor. 2016;10:756–64. 10.1111/crj.12283. 10.1111/crj.12283 25764275

[32] Holter JC, Müller F, Bjørang O, Samdal HH, Marthinsen JB, Jenum PA, et al. Etiology of community-acquired pneumonia and diagnostic yields of Microbiological methods: a 3-year prospective study. Nor. 2015;15:64. 10.1186/s12879-015-0803-5. 10.1186/s12879-015-0803-5 PMC4334764 25887603

[33] Hansen K, Yamba Yamba L, Wasserstrom L, et al. Exploring the microbial landscape: Uncovering the pathogens associated with community-acquired pneumonia in hospitalized patients. Front Public Health. 2023;11:1258981. 10.3389/fpubh.2023.1258981. 38152664 10.3389/fpubh.2023.1258981 PMC10752608

[34] Sellarès-Nadal J, Burgos J, Martín-Gómez MT, et al. Community-acquired pneumonia in hospitalised patients: changes in aetiology, clinical presentation, and severity outcomes in a 10-year period. Ann Med. 2022;54(1):3052–9. 10.1080/07853890.2022.2138529. 36331267 10.1080/07853890.2022.2138529 PMC9639470

[35] Lorentzen MH, Rosenvinge FS, Lassen AT, et al. Empirical antibiotic treatment for community-acquired pneumonia and accuracy for Legionella pneumophila, Mycoplasma pneumoniae, and Clamydophila pneumoniae: a descriptive cross-sectional study of adult patients in the emergency department. BMC Infect Dis. 2023;23(1):580. Published 2023 Sep 5. 10.1186/s12879-023-08565-6 10.1186/s12879-023-08565-6 PMC10481610 37670282

[36] Shoar S, Musher DM. Etiology of community-acquired pneumonia in adults: a systematic review 2020;12:11. 10.1186/s41479-020-00074-3 10.1186/s41479-020-00074-3 PMC7533148 33024653

[37] Braeken DCW, Essig A, Panning M, Hoerster R, Nawrocki M, Dalhoff K et al. Shift in bacterial etiology from the CAPNETZ cohort in patients with community-acquired pneumonia: data over more than a decade 2021;49:533–7. 10.1007/s15010-021-01605-w 10.1007/s15010-021-01605-w PMC8159805 33774804

[38] Mulcahy ME, McLoughlin RM, Staphylococcus aureus and, Influenza A, Virus. Partners in Coinfection 2016;7:e02068-16. 10.1128/mBio.02068-16 10.1128/mBio.02068-16 PMC5156308 27965455

[39] Waagsbø B, Tranung M, Damås JK, Heggelund L. Antimicrobial therapy of community-acquired pneumonia during stewardship efforts and a coronavirus pandemic: an observational study 2022;22:379. 10.1186/s12890-022-02178-6 10.1186/s12890-022-02178-6 PMC9569007 36242006

[40] Woodhead M. Community-acquired pneumonia in Europe: causative pathogens and resistance patterns 2002;36:s20–27s. 10.1183/09031936.02.00702002 10.1183/09031936.02.00702002 12168744

[41] Strålin K, Söderquist B. Staphylococcus aureus in community-acquired pneumonia 2006;130:623. 10.1378/chest.130.2.623-a 10.1378/chest.130.2.623-a 16899873

[42] Blasi F, Garau J, Medina J, Ávila M, McBride K, Ostermann H. Current management of patients hospitalized with community-acquired pneumonia across europe: outcomes from REACH 2013;14:44. 10.1186/1465-9921-14-44 10.1186/1465-9921-14-44 PMC3644236 23586347

[43] Costantini E, Allara E, Patrucco F, Faggiano F, Hamid F, Balbo PE. Adherence to guidelines for hospitalized community-acquired pneumonia over time and its impact on health outcomes and mortality 2016;11:929–40. 10.1007/s11739-016-1445-3 10.1007/s11739-016-1445-3 27098057

[44] Fally M, Diernaes E, Israelsen S, Tarp B, Benfield T, Kolte L, et al. The impact of a stewardship program on antibiotic administration in community-acquired pneumonia: results from an observational before-after study 2021;103:208–13. 10.1016/j.ijid.2020.11.172 10.1016/j.ijid.2020.11.172 33232831

[45] Triantafyllidis C, Kapordelis V, Papaetis GS, Orphanidou D, Apostolidou M, Nikolopoulos I, et al. Guidelines adherence for patients with community acquired pneumonia in a. Greek Hosp. 2012;16:1–9. 22338542

[46] Trent SA, Jarou ZJ, Havranek EP, Ginde AA, Haukoos JS. Variation in emergency department adherence to treatment guidelines for inpatient pneumonia and sepsis: A retrospective. Cohort Study. 2019;26:908–20. 10.1111/acem.13639. 10.1111/acem.13639 PMC7676280 30343515

[47] Pascual Guardia S, Marin-Corral J, Carugati M, Aliberti S, Sibila O, Sanz F, et al. International guideline concordance of empiric antibiotic use in community-acquired pneumonia 2020;56:4669. 10.1183/13993003.congress-2020.4669

[48] Berild AG, Erichsen D, Berild D. Treatment of community-acquired pneumonia 2018;138. 10.4045/tidsskr.17.1115 10.4045/tidsskr.17.1115 30497244

