# Supplementary material for: Microbiological findings and antibiotic treatment in community-acquired pneumonia: a retrospective cohort study
Source: BMC Infect Dis. 2026 Jan 29;26:428. doi: 10.1186/s12879-025-12510-0 (PMC12924426; doi:10.1186/s12879-025-12510-0)
Supplement: Supplementary file 1 — Supplementary Material 1 [file 12879_2025_12510_MOESM1_ESM.docx]

# Supplementary

| Table S1: Guidelines for community-acquired pneumonia | | |  | |
| --- | --- | --- | --- | --- |
|  | **Danish National Guideline** | **North Denmark Regional Guideline** | |  |
| Mild CAP^1^ | Penicillin 1 MIU^2^ | Penicillin 1 MIU | |  |
| Moderate CAP | Penicillin 2 MIU + Clarithromycin | Penicillin 2 MIU + Clarithromycin | |  |
| Severe CAP | Piperacillin/tazobactam + Clarithromycin | Piperacillin/tazobactam + Clarithromycin | |  |
| COPD^3^ patients with mild CAP | Piperacillin/tazobactam + a macrolide | Amoxicillin/clavulanic acid | |  |
| COPD patients with moderate CAP |  | Amoxicillin/clavulanic acid + Clarithromycin | |  |
| COPD patients with severe CAP |  | Piperacillin/tazobactam + Clarithromycin | |  |
| 1) Community-acquired pneumonia  2) Million International Units  3) Chronic obstructive pulmonary disease | | | | |

| Table S2: Microbiological tests | | |  |
| --- | --- | --- | --- |
| Microbiological test | | **Tests performed**  **n (%)** | **Tests positive**  **n (%)** |
| Blood cultures | | 319 (87%) | 15 (5%) |
| Sputum cultures | | 129 (35%) | 40 (31%) |
| Tracheal aspirate cultures | | 3 (0.8%) | 2 (67%) |
| Bronchoalveolar lavage fluid cultures | | 3 (0.8%) | 0 (0%) |
| PCR for atypical pathogens^1^ | 47 (13%) | | 2 (4%) |
| Pneumococcal urinary antigen test | 14 (4%) | | 0 (0%) |
| *Legionella pneumophila* urinary antigen test | 14 (4%) | | 1 (7%) |
| Point of care virus PCR test^2^ | 332 (91%) | | 27 (8%) |
| 1) PCR: polymerase chain reaction; atypical pathogens: *C. pneumoniae, C. psittaci, L. pneumophila and M. pneumoniae*  2) Tests for respiratory syncytial virus (RSV), influenza A virus, influenza B virus and SARS-CoV-2 (COVID-19). | | | |
